# Choice of cystometric technique impacts detrusor contractile dynamics in wistar rats

**DOI:** 10.14814/phy2.14724

**Published:** 2021-01-19

**Authors:** Daniel Medina‐Aguinaga, Robert F. Hoey, Alvaro Munoz, Moises Altamira‐Camacho, Jose L. Quintanar, Charles H. Hubscher

**Affiliations:** ^1^ Department of Anatomical Sciences & Neurobiology University of Louisville Louisville KY USA; ^2^ Department of Physiology & Pharmacology UAA Aguascalientes Mexico; ^3^ Department of Foundations of Knowledge Centro Universitario del Norte University of Guadalajara Colotlan Mexico; ^4^ Kentucky Spinal Cord Research Center Louisville KY USA

**Keywords:** bladder, cystometrogram, external urethral sphincter, micturition, urodynamics

## Abstract

The objective of the current animal study was to investigate factors contributing to the different phases of the cystometrogram (CMG) in order to address disparities in research data reported in the current literature. Three experiments in 20 female Wistar rats were designed to investigate (1) the effects of anesthesia on the contractile pattern of the bladder during micturition; (2) the impact of the physical characteristics of the CMG technique upon the accuracy of intra‐vesical pressure recordings; and (3) identification of physiological and methodological factors associated with the emptying and rebound phases during CMG. Variables tested included awake versus urethane‐anesthetized conditions, use of a single catheter for both filling and intra‐vesical pressure (Pves) recording versus a separate two catheter approach, and comparisons between ureter, bladder dome, and urethral catheter placements. Both awake and anesthetized conditions contributed to variations in the shape and magnitude of the CMG pressure curves. In addition, catheter size, acute incision of the bladder dome for catheter placement, use of the same catheter for filling and Pves recordings, as well as the placement and positioning of the tubing, all contributed to alterations of the physiological properties and characteristic of the various CMG phases, including the frequent occurrence of an artificial rebound during the third phase of micturition. The present results demonstrate how different experimental conditions lead not only to variability in Pves curves, but consistency of the measurements as well, which needs to be accounted for when interpreting CMG outcome data.

## Graphical abstract

The objective of the current animal study was to investigate factors contributing to the different phases of the cystometrogram in order to address disparities in research data reported in the current literature. The present results demonstrate how different experimental conditions lead not only to variability in intra‐vesical pressure curves, but consistency of the measurements as well, which needs to be accounted for when interpreting cystometry outcome data.

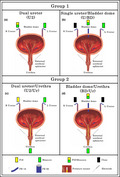

**Abbreviations**
BD: Bladder dome
CMG: Cystometrogram
CP: Closing pressure
EMG: Electromyography
EO‐RP: Early onset rebound phase
EUS: External urethral sphincter
HFPO: High frequency pressure oscillations
ICI: Inter‐contraction interval
LUT: lower urinary tract
PC‐RP: Post‐closure rebound phase
Pves: Intra‐vesical pressure
U: Single ureter
U2: Double ureter
Ur: Urethra

## 1 INTRODUCTION

The non‐stop transvesical cystometrogram (CMG), described in 1986 by Maggi et al. in urethane anesthetized rats (Maggi et al., 1986), has become one of the most important research tools to advance our knowledge of the physiology, pathology, and pharmacology of the lower urinary tract (LUT) (Andersson et al., 2011). Three phases of the CMG include an initial rise in intra‐vesical pressure (Pves) generated by the detrusor's contraction under isovolumetric conditions, an emptying phase identified by phasic contractions of the external urethral sphincter (EUS) which provokes sudden and rhythmic Pves increases known as high frequency pressure oscillations (HFPO) with concurrent loss of pressure during the expulsion of urine (which generates a plateau shape in the Pves record), and a third phase characterized by a Pves increase known as rebound (Andersson et al., 2011; Maggi et al., 1986; Wu et al., 2018). The incidence and contributing factors to the rebound phase have yet to be elucidated, although it has been proposed to be a closing pressure spike (Fraser et al., 2020). In addition, CMG recordings are known to be disrupted by the type of anesthesia used including its route of administration (Cannon & Damaser, 2001; Chang & Havton, 2008; He et al., 2020) as well as the physical properties of the set‐up (tubing size, pump speed), which can induce systematic and random observational errors (Hogan et al., 2012; Lotze, 2005). The objective of the current study was to (a) investigate the effect of catheter implantation and urethane anesthesia on the contractile pattern of the bladder during voiding; (b) determine the role of the physical characteristics of the approach toward the accuracy of the Pves measurement; and (c) identify potential physiological and methodological factors associated with post‐micturition rebound in the rat animal model.

## 2 MATERIALS AND METHODS

### 2.1 Animals

All animal procedures were performed according to the NIH Guide for the Care and Use of Laboratory animals. All protocols were reviewed and approved by the Institutional Animal Care and Use Committee at the University of Louisville, as well as, the Institutional Welfare Regulations of the University Autonomous of Aguascalientes. Twenty female Wistar rats, weighing 250–300 g, were used in three separate experiments. Experiment 1 (*n* = 4) was performed in the Laboratory of Neurophysiology at the University Autonomous of Aguascalientes. Experiments 2 (*n* = 6) and 3 (*n* = 5/group, total *n* = 10) were conducted in the Department of Anatomical Sciences & Neurobiology at the University of Louisville. Rats were kept under a 12‐h light/dark cycle with regulated controlled temperature and humidity. Food (Purina rat chow) and water were available ad libitum.

### 2.2 Anesthesia and post‐surgical care medication

Catheter implantation protocols for Experiment 1 at University Autonomous of Aguascalientes utilized tiletamine and zolazepam (1:1 mixture, 0.4 mg/kg IM, Virbac, Milan, Italy) for surgical anesthesia. Post‐surgical care involved a single injection of amikacin (20 mg/kg IM) and tramadol (4 mg/kg IM) as a prophylactic and analgesic measure. Terminal anesthetized testing utilized urethane (1.2 g/kg SC, Sigma‐Aldrich). Experiments 2 and 3 were non‐survival procedures conducted at the University of Louisville and therefore all procedures were performed under urethane anesthesia only (1.2 g/kg SC, Sigma‐Aldrich).

### 2.3 Bladder dome catheterization surgery

After achieving surgical depth of anesthesia, the urinary bladder was exposed via a midline laparotomy. Cutaneous and fascia layers were separated using sharp dissection whereas the abdominal muscles were separated at the linea alba via blunt dissection. A heat‐flared PE‐50 catheter for Experiment 1 and a PE‐60 catheter for Experiments 2 and 3 were inserted through the bladder dome and secured with a silk ligature (6–0). The catheter and electromyography (EMG) wires (see below) were then tunneled under the skin to the dorsal neck region for externalization. The muscular and cutaneous layers were closed with 4–0 silk suture. Recovering animals (Experiment 1) received post‐care medications (see above) and recovered for 24 h.

### 2.4 Ureter catheterization for non‐survival surgery

A midline laparotomy was performed to expose the urinary bladder and associated anatomy. To expose the ureters, the bladder was moved aside using a 4–0 silk suture attached to the round ligament. For Experiment 2, the left ureter was exposed with blunt dissection and a PE‐10 tubing (pre‐filled with saline) inserted and secured once the end was inside the urinary bladder. For Experiment 3, the left and right ureters were exposed and catheterized, and the tubing secured with silk suture (6–0). The proximal end of the ureters was not closed to allow for drainage of urine into the abdominal cavity. The muscular layer and skin were closed with 4–0 nylon suture. The accurate placement of the catheter into the bladder was visually confirmed at the end of the experiment by opening the bladder.

### 2.5 Wire implantation for external urethral sphincter electromyography (EUS‐EMG)

The urethra was exposed via a midline abdominal incision and blunt dissection with cotton tips. A pair of electrodes (stainless steel coated, 0.002’’, A‐M system, Sequim, WA, USA) was sutured bilaterally in the inguinal ligament and the stripped tip poked into the proximal third of the EUS at 11 and 1 o'clock positions (3 to 5 mm from the distal limit of the bladder neck; note that it was not necessary to cut the pubic symphysis). A third electrode was sutured to the abdominal wall as a reference electrode (stainless steel coated, 0.003’’, A‐M system). Wires were then tunneled under the skin and externalized at the dorsal neck region. The correct placement of the wires was verified by evoking the guarding reflex via compression of the suprapubic abdomen (D’Amico & Collins, 2012) and by direct visualization during necropsy.

### 2.6 Experimental procedures

#### 2.6.1 Experiment 1

After recovering for 24 h, a CMG on fully awake and freely moving rats was conducted in a metabolic cage. The catheter was connected via a three‐way connector to an infusion pump for saline infusion (Braintree Scientific, Braintree, MA, USA), and a pressure transducer (Biopac Systems Inc., Santa Barbara, CA) for bladder pressure recording. Voiding responses were elicited by continuously infusing saline (0.9% NaCl) at a rate of 0.12 ml/min at room temperature (approximately 22°C). The same solution, temperature, and infusion rate were used in all three experiments. Urine was collected in a container coupled to a weight transducer (Biopac Systems Inc.). The cystometric evaluation was performed until 10 micturition cycles were recorded. After an additional 24 h, the same rats were anesthetized with 1.2 g/kg s.c. of urethane and a CMG test was once again performed. The CMG parameters analyzed included inter‐contraction interval, baseline bladder pressure, maximal bladder pressure, and voided volume.

#### 2.6.2 Experiment 2

After induction of anesthesia with urethane, the left ureter was catheterized, and the EUS‐EMG electrodes implanted (per above). The bladder catheter was then connected via a three‐way connector to an infusion pump for saline infusion (Braintree Scientific, Braintree, MA, USA) and a pressure transducer (World Precision Instruments [WPI, LLC]; Sarasota, FL, USA) for bladder pressure recording. The three EUS‐EMG electrodes were connected to a differential amplifier (A‐M Systems, Sequim, WA, USA). The animal was placed in a supine position on a surgical table in a reverse Trendelenburg position and a container coupled to a weight transducer (WPI, LLC) was placed under the rat for urine collection and measurement. Ten urination cycles were elicited with saline (rate: 0.12 ml/min). After the recording of 10 fill/void cycles, the abdominal wall was re‐opened and a flared PE‐60 catheter inserted into the bladder dome as described above, and the opening re‐closed. The ureter catheter was sealed and replaced with the dome catheter by connection to the pressure transducer and pump for the collection of 10 more fill‐void cycles. CMG parameters analyzed included baseline basal pressure, inter‐contraction interval, bursting period duration, void pressure, and maximal bladder pressure.

#### 2.6.3 Experiment 3

**FIGURE 1:**
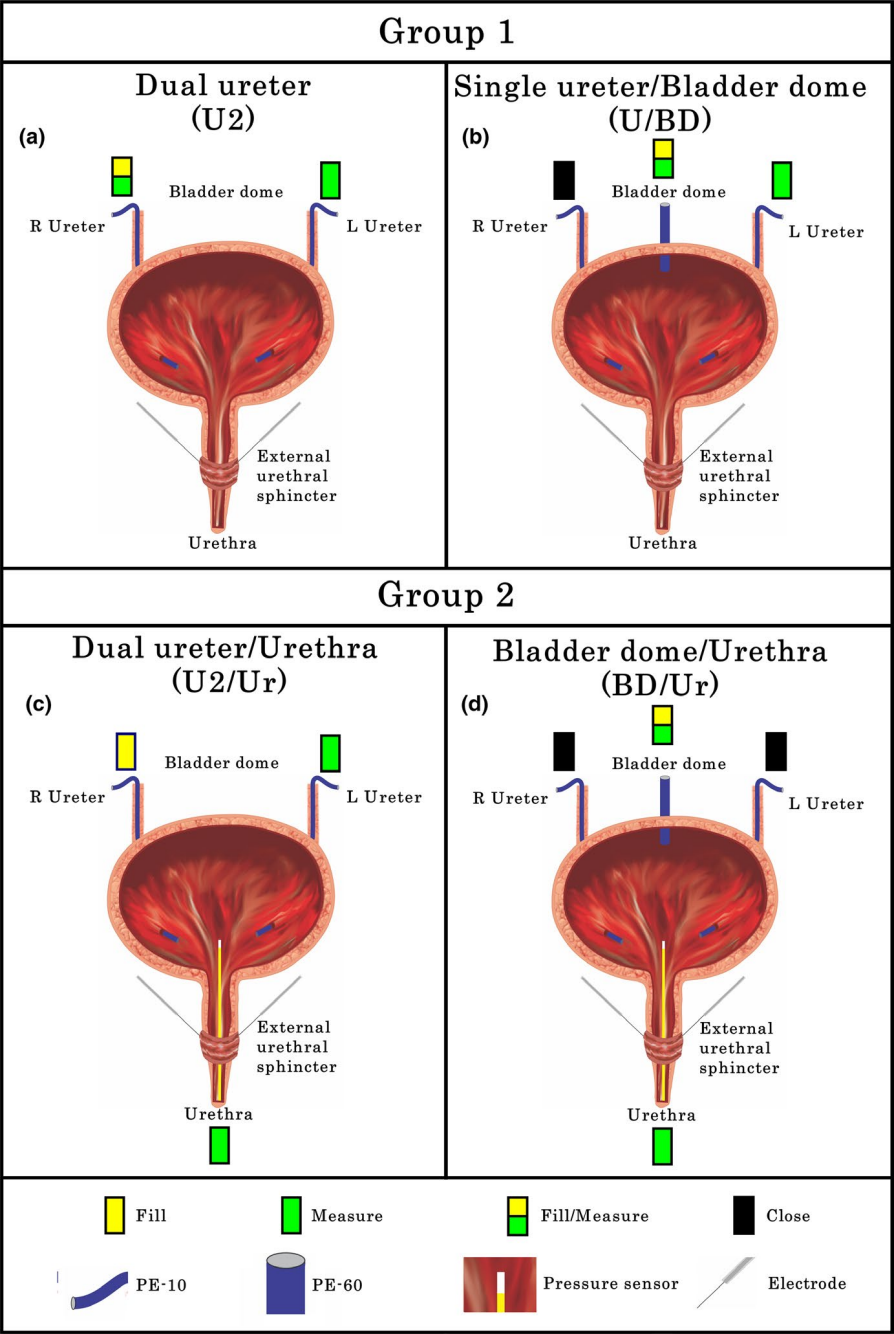
Different configurations for non‐stop cystometrograms used in Experiment 3. (a) Two ureter catheter (U2) approach: PE‐10 tubing was placed in the right ureter for saline infusion and Pves measurement. A second PE‐10 tubing in the left ureter was used for Pves measurement only. (b) Single ureter (U) and bladder dome (BD) catheter (U/BD) approach: A PE‐60 catheter was placed in the bladder dome for filling and Pves measurement. A PE‐10 catheter in the left ureter was used for Pves measurement and the right ureter catheter was closed. (c) U2 with urethra catheter (U2/Ur) approach: A PE‐10 tubing was placed in the right ureter for filling and PE‐10 in left ureter for Pves measurement. Additionally, a pressure transducer was placed into the urinary bladder through the urethra. (d) BD with urethra catheter (BD/Ur) approach: A PE‐60 catheter was placed in the bladder dome for filling and Pves measurement and both ureteral catheters were closed. A pressure transducer was placed into the urinary bladder through the urethra

Four CMG configurations, illustrated in Figure 1, having single, double or triple urinary bladder catheterizations, were utilized. Configurations are divided into two groups based upon absence/presence of a urethral catheter.

##### Group 1

A double ureter (U2) configuration (Figure 1(a)) was set up for testing of 10 void cycles whereby separate channels for filling and Pves measurement can be compared with the approach used in Experiment 2 (a single catheter used for both filling and Pves measurements). After U2 testing, a bladder dome catheter was added (Figure 1(b)) and 10 more fill‐void cycles performed for within animal comparisons.

##### Group 2

Starting with the U2 configuration (Figure 1(a)), 10 fill/void cycles were recorded. A probe with a pressure transducer in the tip (Millar Mikro‐tip Catheter Transducer, AD Instruments. 2.5F) was then placed directly into the urinary bladder through the urethra (Configuration U2/Ur, Figure 1(c)), and 10 more fill‐void cycles performed. After removal of the urethral catheter, a PE‐60 catheter was inserted through the bladder dome and 10 more void cycles were recorded (Configuration U/BD, Figure 1(b)). Lastly, the urethral pressure transducer was reinserted into the bladder (Configuration BD/Ur, Figure 1(d)) and ten additional cycles were recorded. In a subset of 3 rats, after 5 cycle recordings, the dome catheter was rotated 180° and pushed approximately 3–4 mm in the direction of but not into the bladder neck in order to mimic deformation of the bladder dome and contact of the intravesical catheter tip with the bladder wall, which are common occurrences during cystometry. Five more fill‐void cycles were then recorded.

#### 2.6.4 Euthanasia

Animals were euthanized by urethane overdose after all experimental procedures.

### 2.7 Data collection and analysis

Data in Experiment 1 were acquired and quantified with AcqKnowledge software (Biopac Systems Inc.). Experiments 2 and 3 utilized Spike2 software (CED, Cambridge, UK) for acquisition and quantification. A pressure calibration was carried out using the PRSIM pressure simulator (WPI. Sensitivity: 5 µV/V/mm Hg). This methodology does not measure the added pressure generated by the saline pump. In all the experiments, the data acquisition rate was 10 KHz. CMG and pressure traces were filtered using a 20 Hz low‐pass filter and smoothing process. EMG traces had a band pass filter applied (signals between 50 and 500 Hz are allowed through), with a notch filter at 60 Hz to eliminate electronic interference. EMG signals were amplified (1,000×).

For analysis, the last three of the 10 micturition cycles per rat were used for consistency (last 3 of 5 in Group 3 Experiment 3 rats) and to assure stability of the continuous fill‐void cycles. Statistical analysis was performed using paired T‐tests (Experiments 1 and 2) and linear regression analysis (Experiment 3) with significance level set at *p* ≤ 0.05 (Prism 7, GraphPad, San Diego, CA). Data are shown throughout the manuscript as mean ± SEM.

## 3 RESULTS

### 3.1 Experiment 1

**FIGURE 2:**
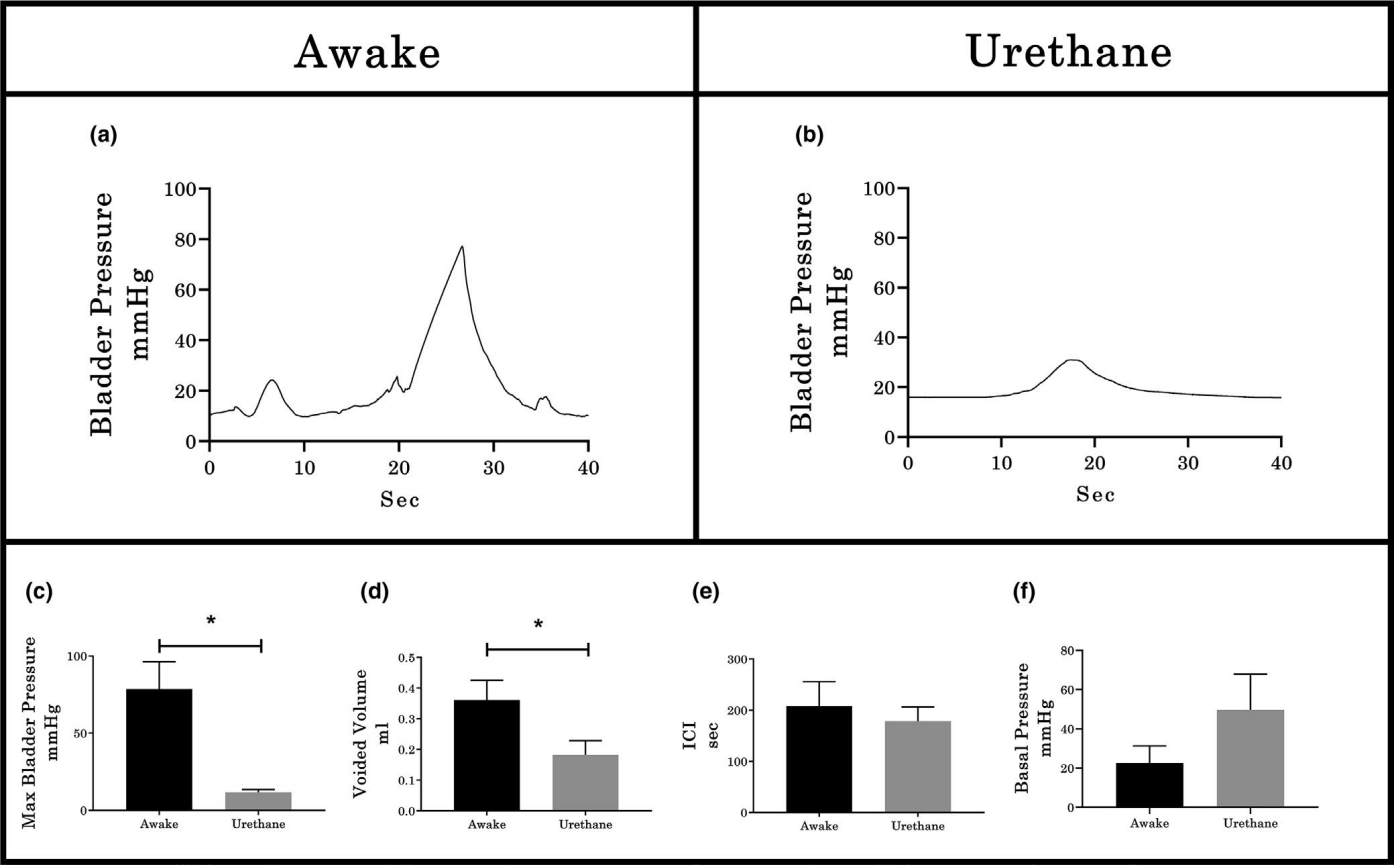
Examples of CMG in awake and urethane‐anesthetized rats. (a) In awake animals, 24 hours post‐catheter placement, the contractile curves were smooth (with no HFPO or appreciable rebound) with high maximal bladder pressure. (b) After an additional 24 hours, the same animals under urethane anesthesia still had smooth contractile curves but with lower amplitude. Histograms represent the quantification of cystometrographic variables showing that urethane anesthesia results in significantly reduced maximum bladder pressure (c) and void volume (d), but no effect on either intercontractile interval (e; ICI) or basal pressure (f). Values represent mean ± SEM; * indicates *p* < .05 between groups (paired *T*‐test)

Performing CMG using a single catheter approach through the bladder dome for both filling and Pves measurement in awake rats (*n* = 4) at 24 h post implantation reveal mean basal pressure at 10 cm H_2_O (equivalent to 7.356 mm Hg) and smooth contractile pressure curves during the void period, without any HFPO or opening and closing pressure spikes (see typical example in Figure 2(a)). Note that baseline pressures no greater than 10 cmH_2_O are recommended (Andersson et al., 2011). A CMG performed in the same rats under urethane anesthesia after an additional 24‐hour period also had high basal pressure and a smooth contractile curve (see typical example in Figure 2(b)). Quantification of all CMG parameters indicates significant suppression of maximal bladder pressure (*p* < 0.05, Figure 2(c)) and voided volume (*p* < 0.05, Figure 2(d)) with urethane anesthesia, but no differences in either the inter‐contraction interval (Figure 2(e)) or basal pressure (Figure 2(f)). After euthanasia, different degrees of obstruction (blood clots and crystals) were observed at the tip of the catheters of all four animals.

### 3.2 Experiment 2

**FIGURE 3:**
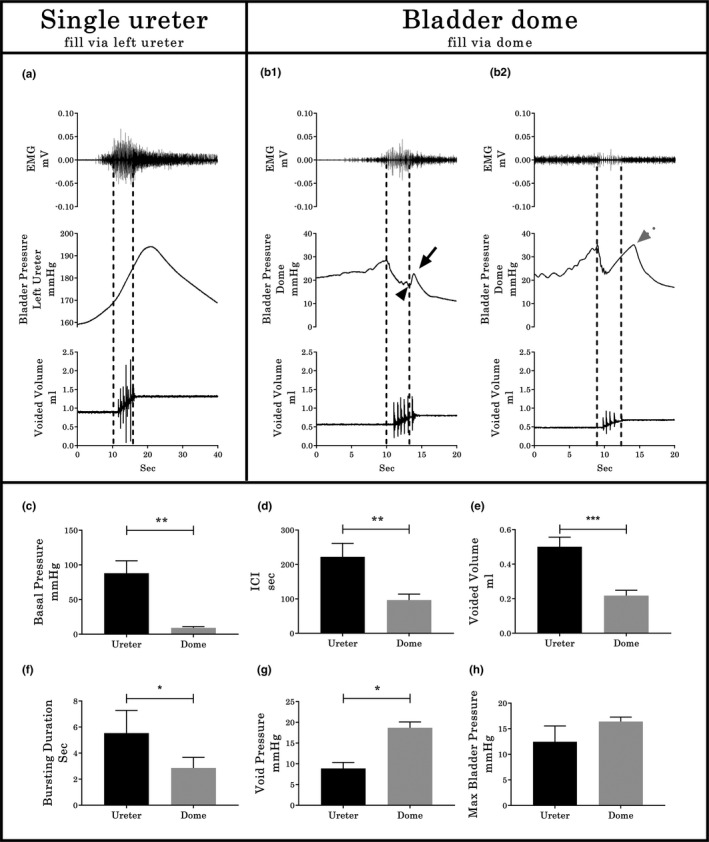
Cystometrographic and electromyographic differences between ureter or bladder dome catheterization sites. (a) CMG/EUS‐EMG traces in rats where the bladder was filled via the ureter and the bladder remained intact. (b) CMG/EUS‐EMG traces in rats after subsequent catheterization via an incision of the vesical dome. In both a and b, Pves is recorded through the same catheter used for filling. Note the two different CMG/EUS‐EMG patterns (b_1_ and b_2_) for the fill/Pves performed through the bladder dome. Differences found in the baseline pressure (c), ICI (d), voided volume (e), EMG bursting time (f), void pressure (g), and maximal bladder pressure (h) between groups are shown (*n* = 6, pair‐wise comparisons). In b_1_, the black arrowhead indicates the closing pressure and the black arrow the post‐closing pressure rebound phase. In b_2_, the dotted gray arrow indicates an early onset rebound phase. Values represent mean ± SD. * indicates *p* < .05; ** indicates *p* < .01; *** indicates *p* < .001 by paired *T*‐test

In all six urethane‐anesthetized rats tested, the pressure curve recorded during cystometry using a single catheter placed in one ureter for both filling and Pves measurement was smooth but had a high‐pressure baseline (typical example provided in Figure 3(a)). Subsequent catheterization through the bladder dome during the same terminal experiment (Figure 3(b)) resulted in a significantly lower baseline pressure (*p* < 0.01, Figure 3(c)) and the presence of an opening pressure spike, HFPO during the expulsion period, and a closing pressure spike. Note that the CMG/EUS‐EMG performed through the bladder dome exhibited two different patterns. The first pattern (50% of total) consisted of similar duration of bursting, HFPO and urine flow (typical example demarcated by dotted lines in Figure 3(b1)). The second pattern (50% of total) presents similar duration of bursting and urine flow but shorter HFPO duration (5.55 ± 0.63 vs 3.11 ± 0.61 seconds, *p* = 0.01, demarcated by dotted lines in Figure 3(b2)). These patterns were further examined in Experiment 3 (shown using simultaneous urethral recordings to reflect differences in the timing of the CMG Phase 3 rebound onset). For Experiment 2, the inter‐contraction interval (*p* < 0.01, Figure 3(d)), voided volume (*p* < 0.001, Figure 3(e)), and the EUS bursting time (*p* < 0.05, Figure 3(g)) were all significantly lower with bladder catheterization through the dome, although, the opening pressure was significantly increased (*p* < 0.05, Figure 3(g)). No statistical differences were found in the maximal bladder pressure between catheterization methods (see Figure 3(h)).

### 3.3 Experiment 3

#### 3.3.1 Group1 without urethral recording (*n* = 5)

**FIGURE 4:**
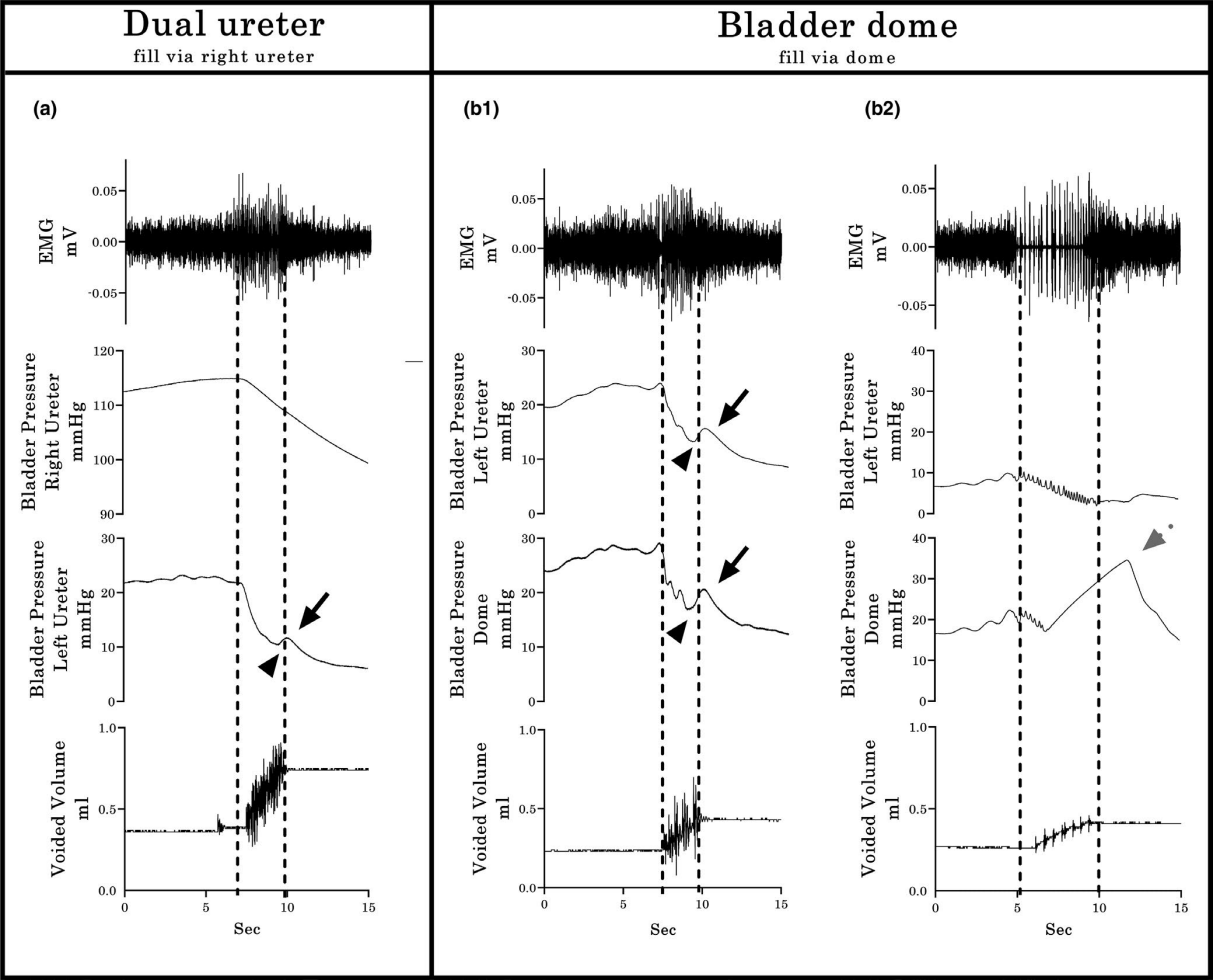
Typical Group 1 examples of bladder pressure curves recorded by different cystometrographic techniques. (a) The dual ureter shows a dimorphic pattern: The Pves shape obtained by the right catheter, which was used for filling and measurement, has a continuous descending pressure, even during expulsion of urine, without HFPO in the plateau phase and rebound effects. The second pattern, obtained simultaneously through the left catheter, which was used only for Pves measurement, includes the second CMG phase plateau with HFPO’s and similar timing in relation to bursting time and urine flow (dashed lines), with small amplitude rebound events in the third phase (black solid arrows indicate rebound phase of the pressure curve; arrowheads indicate closing pressure). (b_1_ & b_2_) Filling through a dome‐placed catheter shows two distinct response patterns (as in Experiment 2; see Figure 3(b)). However, in this experiment, the simultaneous Pves only ureter recordings indicate a profound post‐void pressure increase coupled with a reduced HFPO duration within the pressure curve recorded with the dome catheter (gray dotted arrow in b_2_), indicating early onset of the rebound phase. Shown from top to bottom are the EMG’s of the external urethral sphincter (EUS), the Pves curves obtained by a catheter (in a, right ureter for both fill and Pves recording and left ureter from Pves‐measure only; in b, left ureter for Pves‐measure only and bladder dome for fill and Pves record), and the volume captured during the voids. Dashed vertical lines show the onset and offset of EUS‐EMG bursting, thereby highlighting the temporal relationship to changes in the other dependent measures

In five urethane‐anesthetized female rats having the dual ureter configuration (U2, Figure 1(a)), pressure curves were recorded simultaneously from both the catheter being used for filling (traditional way with single catheter technique) as well as the other ureter catheter not being used for filling (Pves measurement only). Whereas the pressure curves from the catheter used for both fill and Pves measurements showed high baseline pressure and smooth pressure curves during micturition, the other Pves‐only catheter had a low baseline pressure and showed the plateau with HFPO during micturition, and a small rise of Pves in the third phase (i.e., rebound, Figure 4(a)), indicating a smoothing effect upon the pressure curve when simultaneously filling. Upon subsequent placement of a catheter through the bladder dome (U2/BD configuration; Figure 1(b)), 40% of the CMGs showed similar pressure curves in the ureter versus dome (Figure 4(b1)), which were comparable with the prior record‐only ureter pressure curve (re Figure 4(a)). However, although the other 60% showed the same ureter CMG pattern (Figure 4(b2)), the dome CMG had a pattern like the one seen in Experiment 2 (example Figure 3(b2)).

#### 3.3.2 Group 2 using urethral recording (*n* = 5)

**FIGURE 5:**
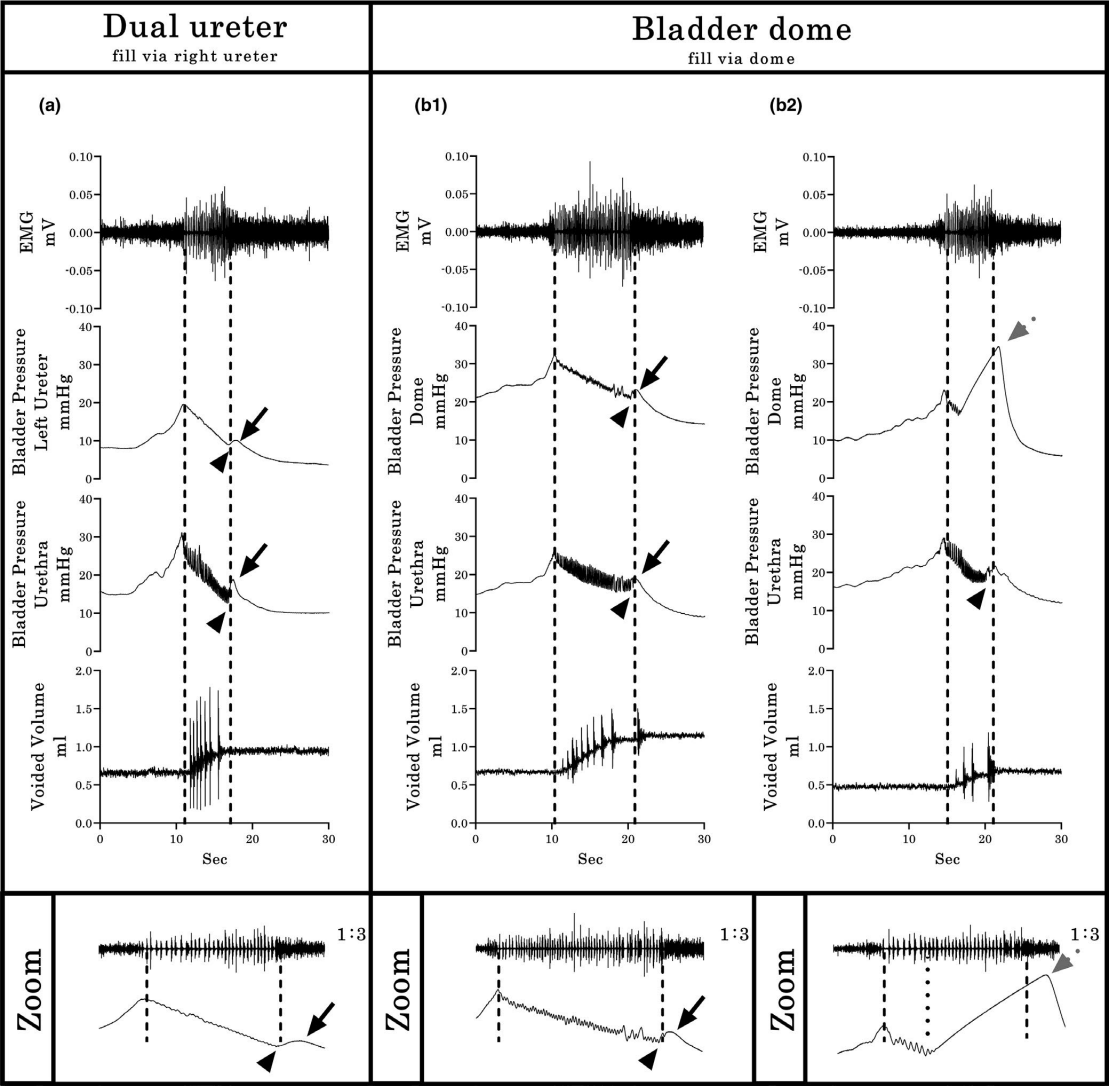
Typical Group 2 examples of bladder pressure curves recorded by different cystometrographic techniques. Shown from top to bottom are the EMG’s of the external urethral sphincter (EUS), the Pves curves obtained by a catheter (ureteral from Pves‐only catheter in a or bladder dome from same fill catheter in b), the Pves recorded by a pressure transducer placed in the bladder through the urethra, and the volume captured during the voids. Dashed vertical lines show the onset and offset of EUS‐EMG bursting, thereby highlighting the temporal relationship to changes in the other dependent measures. At the bottom of each column, a zoomed (1:3) image of the EUS‐EMG and corresponding CMG traces are shown, indicating with black arrowheads the closing pressure (CP) and with black arrows the rebound phase of the pressure curve. (a) The dual ureter with urethra placement shows similar timing in relation to bursting time and urine flow, with a small amplitude post‐closure rebound phase (PC‐RP). (b_1_ & b_2_) Filling through a dome‐placed catheter shows two distinct response patterns. The first pattern (b_1_) consists of both Pves recordings, urethral and dome, with identical PC‐RP patterns as with the ureter filling in a. Importantly, although the second pattern (b_2_) shows the same PC‐RP pattern in the urethral sensor, the dome catheter recording again indicates a profound post‐void pressure increase coupled with a reduced HFPO duration (gray dotted arrow). The dotted line in the zoomed figure of b_2_ highlights the temporal discoordination between the onset of the early onset of the rebound phase (EO‐RP) and the offset of the EUS‐EMG bursting

Simultaneous recording with a catheter inserted through the urethra indicates, as seen with the ureter pressure‐only recording in Group 1 (Figure 4(b2)), an existing temporal discoordination between the onset of the rebound rise in Pves and the offset of the EUS‐EMG bursting in a subset of pressure curve recordings. This discoordination, referred to hereinafter as early onset rebound phase (EO‐RP), differs from the expected outcome of the rebound phase onset upon closure of the EUS (post‐closure rebound phase [PC‐RP]; i.e., upon offset of bursting and concurrent micturition). For the U2/Ur configuration (Figure 1(c)), the pressure curve during micturition had a PC‐RP in the urethra in addition to the ureter CMGs (Figure 5(a)). In contrast, for the BD/Ur configuration (Figure 1(d)), all the animals showed the same PC‐RP in the bladder dome record as well as in the urethral record (Figure 5(b1)). However, in the subset of three animals where the dome catheter was pulled and twisted intentionally after collection of the first 5 of 10 fill‐void cycles, the remaining pressure curves for the dome catheter shifted to the EO‐RP pattern (see typical example in Figure 5(b2)). Note that the burst duration when the urethral catheter is in place is significantly higher (*p* = 0.026; Mean 3.148 s with U2/UR configuration vs 6.128 s with U2 configuration). We hypothesize that the partial obstruction of the urethra due to the pressure sensor is compensated by an increase in the duration of the bursting period without compromising the void efficiency, since no statistical differences were found in the ICI, voided volume, opening pressure, or rebound amplitude between U2 and U2/Ur catheterization methods. In addition, no statistical differences were found in the busting duration, ICI, voided volume, void pressure or rebound amplitude between BD and BD/Ur catheterization methods.

#### 3.3.3 Characterization of CMG/EMG response patterns:

To help elucidate the most accurate shape of the Pves curve, all the recordings from Experiments 2 and 3 (*n* = 16) obtained via the bladder dome were further analyzed. Micturition pressure curves were classified into one of the two different patterns based upon the shape of the Pves wave and the temporal correlation between the bursting period in the EUS and the HFPOs in the Pves pressure wave. The first pattern (PC‐RP; *n* = 8) includes those micturition events with an equal duration for bursting and HFPO, with a brief increase in bladder pressure after EUS closure. The second pattern (EO‐RP; *n* = 8) includes those micturitions where the HFPOs are masked by a pressure artifact during the EUS bursting period (HFPOs are still present in simultaneous urethral recordings; see below). Note that the closing pressure (CP) is the pressure value in the curve that occurs when the sphincter closes (end of bursting). The CP is frequently different from the maximum PC‐RP pressure, which is the highest‐pressure point that is reached after the sphincter has closed.

**FIGURE 6:**
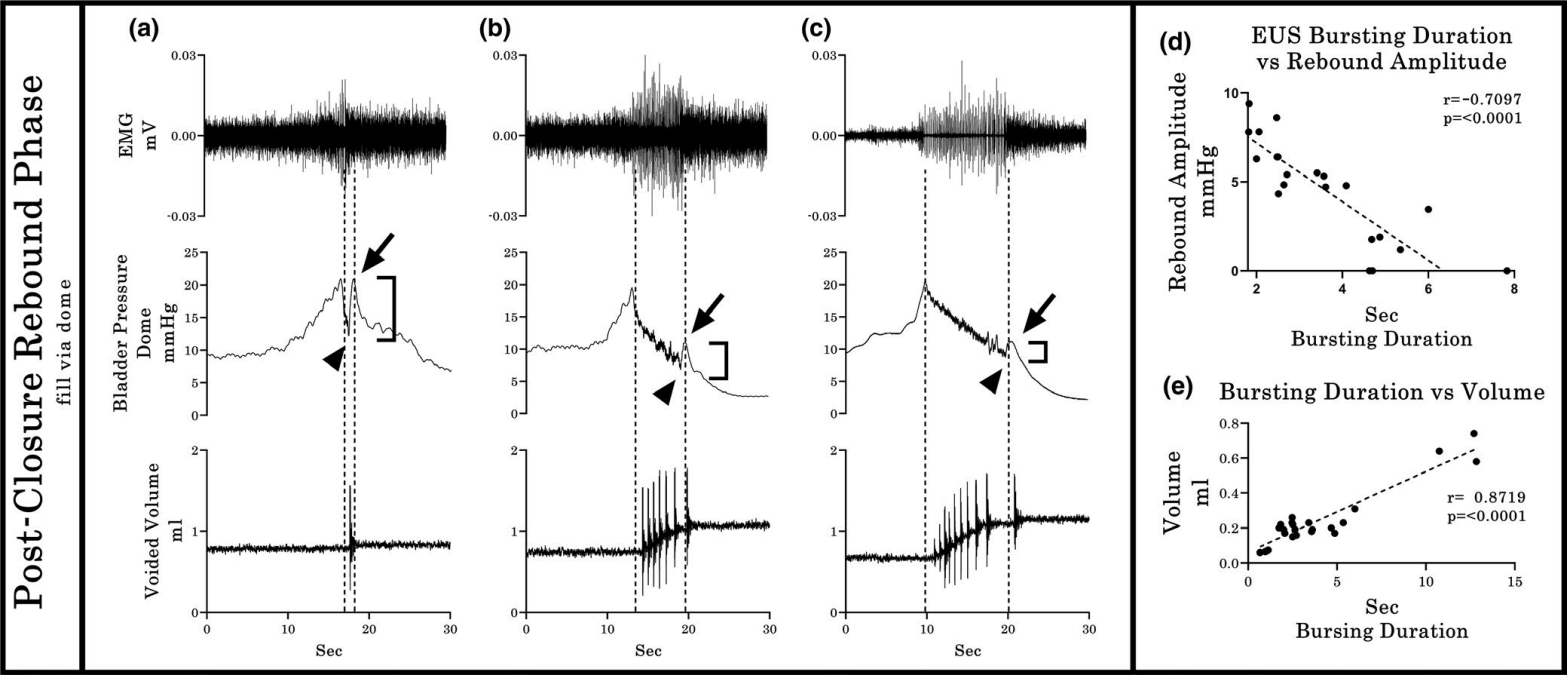
Relationship between EUS burst duration, HFPOs duration, rebound amplitude, and voided volume in the Post‐Closure Rebound Phase group. Typical examples (a–c), showing the variability of Pves amplitude in the third phase of micturition. In the PC‐RP group, the EUS burst duration and Pves rebound amplitude showed a negative relationship (*r* = −0.7097), indicating a shorter burst duration is associated with a larger amplitude rebound (d). At the same time, the EUS bursting duration correlates positively (*r* = 0.8719) with the voided volume in this group (e)

**FIGURE 7:**
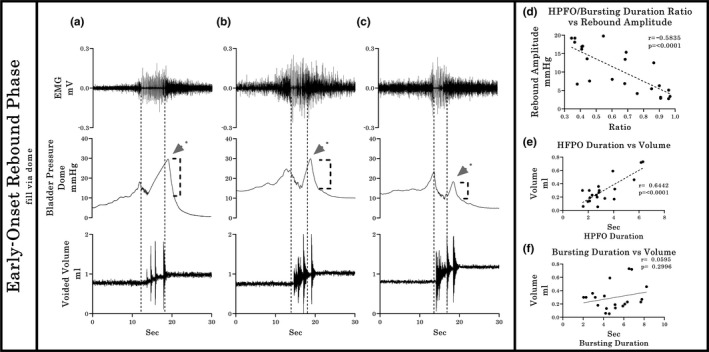
Relationship between EUS burst duration, HFPOs duration, rebound amplitude, and voided volume in the Early Onset—Rebound Phase group. For the EO‐RP patterns (typical examples provided in a–c), a negative correlation (*r* = −0.5835) was found for the Pves rebound amplitude and the Bursting/ HPFO duration ratio, meaning that the earliest occurrences of the onset of the Pves rise in relation with the EUS‐EMG bursting offset correlates with larger maximum rebound amplitude (a). There is also a positive correlation (*r* = 0.6442) between the HFPO duration and the voided volume (e). However, the direct proportionality between the duration of the EUS‐EMG bursting duration and voided volume is lost (f; *p* = 0.2996). The dotted vertical lines show the temporal relationship between the bursting period, the HFPO and the duration of urine flow

Typical examples illustrating comparisons between PC‐RP and EO‐RP response patterns from recordings obtained by the bladder dome catheterization in Experiments 2 and 3 are provided in Figures 6 and 7. Multiple significant correlations were found amongst the various recorded outcome measures. For example, a significant inverse relationship existed between the amplitude of the Pves rebound and the duration of the bursting period (*r* = −0.71, *p* < 0.0001, Figure 6(d)) in PC‐RP responses, indicating that a shorter duration of bursting, detected either indirectly as HPFO or directly as EUS EMG bursting activity, is associated with a larger rebound pressure rise after micturition. In addition, a statistically significant positive correlation (*p* < 0.0001, *r* = 0.8719) was found between the duration of the EUS bursting period and the voided volume in PC‐RP responses (Figure 6(e)), indicating that longer bursting activity correlates with larger volumes. In contrast, for the EO‐RP response patterns, whereas the Pves rebound was negatively correlated with the HPFO/bursting duration ratio (*r* = −0.5835, *p* < 0.0001, Figure 7(d)) and the HFPO duration correlated positively with the voided volume (*r* = 0.6442, *p* < 0.0001, Figure 7(e)), there was no correlation (*r* = 0.0696, *p* = 0.2996, Figure 7(f)) between burst duration and volume. However, there were no significant differences in voided volumes between PC‐RP and EO‐RP groups (0.2449 ± 0.0610 vs 0.2904 ± 0.0714, *p* = 0.6337).

## 4 DISCUSSION

The results of the three current experiments when taken together indicate that multiple variables influence the size, timing, and shape of the intra‐vesical pressure curves during CMG. In the first part of the study, use of urethane suppressed pressure and voiding relative to awake conditions, a finding consistent with other comparable studies and the impact urethane has upon central glutamatergic transmission (Matsuura & Downie, 2000; Morikawa et al., 1989; Yoshiyama et al., 2013) and the mobilization of calcium ions (Maggi et al., 1984). Also, higher basal pressures with urethane relative to awake conditions may be due in part to a reduction in the transversal area of the implanted tubing from clot formation at 48‐h post‐implantation. Use of larger tubing (PE‐60 or 90 instead of PE‐50) for long term awake CMG’s with periodic catheter flushing is recommended (Birder et al., 2007; Gammie et al., 2014; Maggi et al., 1986). Furthermore, to avoid multiple pressure spikes from movement during the filling phase in awake recordings, measuring abdominal pressure and subtracting the activity from the detrusor pressure (Lee et al., 2008) could be employed in future studies.

Although no remarkable changes were found during the filling phase in any of the CMG experiments, multiple differences were found during the emptying phase between experiments. In Experiment 1, a smooth large amplitude contractile curve occurred during emptying (Phase 2), without any Phase 3 pressure rebound. The lack of a pressure decrease and HFPOs (associated with the pumping action of skeletal muscle structures such as the rhabdosphincter (Maggi et al., 1986; Thor & Groat, 2010) and EUS (Chen et al., 2012; Conte et al., 1991; Cruz & Downie, 2005)) following the opening of the urethra is a consistent finding seen in studies carried out in awake animals (Cannon & Damaser, 2001; Conte et al., 1988; Cruz & Downie, 2006; He et al., 2020; Jin et al., 2009). One of the few explanations provided in the literature relates to infusion rate, where higher filling rates are hypothesized to trigger the compensatory HFPO mechanism resulting in more efficient emptying under urethane anesthesia (Cannon & Damaser, 2001; Maggi, Giuliani, et al., 1986). It is worth noting that the same rats tested under urethane‐anesthesia 24‐h later (at 48‐h post‐implantation, Experiment 1) as well as the animals tested with filling through the ureter in Experiment 2 had smooth contractile curves. In addition to infusion rate, the smoothing of the Pves curve may also occur from the use of a single catheter system for simultaneous filling and pressure measurements. The low‐pressure and high flow velocity generated in the small transverse areas of the tubing in combination with the active pumping of saline could act as a mechanical filter, thereby mitigating the effect of the sudden drop of pressure due to the opening of the urethra as well as the small hydraulic forces generated by the rhythmic contraction of the sphincter. Thus, these conduit‐associated phenomena likely contributed to a Pves curve with a continuous ascending phase without HFPO during the expulsion of urine or the rebound component that follows it. Use of a dual catheter system is therefore recommended when possible to avoid the pressure artifacts generated by pumping saline though the same tubing in order to record the low amplitude HFPOs and the plateau (Fraser et al., 2020). For single catheter use, strategies that can be used to enable observation of the HFPO include using a short and rigid catheter (Kontani, Kawabata, 1987, 2009; Kontani, Kobayashi, 1987) or reducing infusion rate (Watanabe & Constantinou, 1996; Watanabe et al., 1997).

For Experiment 2 and 3 CMG recordings from catheters implanted through the dome, the Pves curves generated had HFPOs within the plateau phase as well as a rebound pressure increase (Figure 3(b1) and (b2)). The larger internal diameter of the PE‐60 catheter allowed a more accurate measure of the Pves by reducing the filtering effects of the smaller tubing (Schäfer et al., 2002; Yaksh et al., 1986). The HPFOs were higher in amplitude than those recorded via the ureter, but smaller than the ones recorded with the urethral transducer (see Figure 5). Note that the reduction of the urethral lumen by the pressure transducer also modifies the performance of micturition, the most notable change being the lengthening of the phasic activity of the EUS (Smith et al., 2008). Also, the placement and testing order in the same animals (ureter then dome catheter) could have had an effect and therefore is a potential limitation of the study.

It is important to note that the different CMG approaches in anesthetized animals impacted both the detrusor contraction and the EUS. For example, incision of the bladder dome for catheter insertion induced a significant reduction in the ICI and EUS burst duration, and thus the volume voided (Figure 3(d)–(f)). Although the mechanisms are unclear, endothelial damage has been previously associated with a reduction of the EUS bursting period (Wu et al., 2018) and inflammatory responses to acute catheterization placement have been shown to require 5–7 days to resolve (Matsuura & Downie, 2000; Yaksh et al., 1986). Furthermore, acute release of pro‐inflammatory cytokines, prostaglandins, as well as urothelial neurochemicals have been shown to induce bladder overactivity (Birder, 2006; D’Amico & Collins, 2012; Van Asselt et al., 1995).

During the third and final phase of micturition which begins with the closure of the EUS, an increase followed by a decrease in Pves was detected. Premature closing of the bladder outlet during an active detrusor contraction generates a rise of Pves due to new isovolumetric conditions (Fraser et al., 2020; Groen et al., 1994; Watanabe & Constantinou, 1996). In humans, the premature closure of the bladder outlet has been described as a cause of the after‐contraction phenomenon (Brantley et al., 1964; Zinner et al., 1963), which is considered a normal finding in healthy children. In rats, this Pves increase during the third phase has been labeled in different ways; amplitude of micturition contraction (Maggi et al., 1986), maximum intravesical pressure (Chang & Havton, 2008), peak pressure (Yaksh et al., 1986), maximum pressure (Andersson et al., 2011), and most recently as closing pressure (Fraser et al., 2020; Yoshiyama et al., 2013 In the current study, the term “post‐closure rebound phase” (PC‐RP) is used to refer to this phenomenon for several reasons. First, the micturition contraction cannot be determined in an open system (Fraser et al., 2020), therefore maximal pressure peak very often occurs during the first phase (Cruz & Downie, 2005; Salazar et al., 2019). Second, the closing pressure refers specifically to the pressure value at the moment of sphincter closure (concurrent with the end of bursting in the rat) (Cruz & Downie, 2005; Jin et al., 2014; Vera & Nadelhaft, 2001) which requires simultaneous EMG of the EUS. However, the pressure rise in the third phase of the micturition hits the higher value some seconds after EUS closure (Cheng et al., 1997; Cruz & Downie, 2005; Salazar et al., 2019).

In terms of the Pves peak after EUS closure, which was observed in all configurations tested in Experiments 2 and 3, the amplitude was quite variable, having an inverse relationship with the duration of EUS bursting (Figure 6(d)). The residual volume remaining in the bladder after the micturition is important to consider as an explanation of the variable amplitude. Laplace's Law (Δp = γ(1/R1 + 1/R2), where: Δp = Pves, γ = the detrusor tension, and R1 and R2 are the principal radii of bladder at a specific volume) predicts how the same tension generated by the detrusor can generate different values of pressure depending on the volume of the bladder (Ruarte et al., 2002); however, this variable was not explored as it was beyond the scope of this study. Note that when the bursting activity is longer in duration, as occurs with probe obstruction during urethral catheterization, the flow of urine remains even after the detrusor contraction, eliminating the post‐void isovolumetric contraction and the consequent rebound, giving the impression than the closing pressure and the PC‐RP are the same.

A second pattern of detrusor activity, observed during the third phase of micturition, was termed early onset rebound phase (EO‐RP) to reflect the timing discrepancy between duration of HFPOs and EUS bursting (Figure 7). Several pieces of evidence refute a second detrusor contraction as the source of the Pves increase during the bursting phase. For example, bladder contractions evoking urine expulsion are mostly single and continuous (Cannon & Damaser, 2001; Conte et al., 1991; Fraser et al., 2020; Maggi, Giuliani, et al., 1986; Salazar et al., 2019; Streng et al., 2006) and Pves is unlikely to return to an elevated level due to the loss of volume and the pressure release accompanying an open urethra (Andersson et al., 2011; Fraser et al., 2020; Mastrigt & Griffiths, 1979; Van Asselt et al., 1995; Watanabe & Constantinou, 1996). Additionally, HFPOs are generated by the rhythmic contraction of the EUS (Maggi, Giuliani, et al., 1986; Maggi et al., 1984, 1986; Vera et al., 2001; Vera & Nadelhaft, 2001) and the duration of urine flow and EUS bursting activity are the same (Conte et al., 1991; Kontani et al., 2009; Matsuura & Downie, 2000; Smith et al., 2007; Zhang et al., 2018). Although a Pves increase could be produced by extra‐vesical conditions such as movements and abdominal wall contraction (Fraser et al., 2020; Schäfer et al., 2002; Zinner et al., 1963), these experiments were performed in fully anesthetized animals where the movements were minimum or absent. Also, the contribution of abdominal wall contraction to Pves during the micturition are known (Smith et al., 2007; Zhang et al., 2018) and thus are not likely a factor, as evidenced in other studies (Chang et al., 2007; Cruz & Downie, 2006; Jin et al., 2014; Lee et al., 2008; Salazar et al., 2019).

A more likely source(s) of the second Pves increase during the expulsion phase comes from the setup and/or equipment itself (Schäfer et al., 2002). Factors such as the calibration methodology, amplification and filtering of the pressure transducer signaling, acquisition rate, and the physical aspects concerned with pressure transduction can modify the pressure record (Hundley et al., 2006). The purpose of using two different pressure sensors during the micturition in the current study was to determine the accuracy of each approach to register changes in the Pves. Several current findings and those from other laboratories lead to the conclusion that the EO‐RP is of an artificial nature. First, EO‐RP occurs only in CMG recordings that use a single dome catheter for both filling and measurement (Figures 3(b2), 4(b2), 5(b2), 7(a–c)) (see also 2, 5, 41, 43, 58). Second, the CMG approach using the urethra, ureters or dome with a passive catheter (no infusion through it) have HFPOs timed with burst duration (as seen with the PC‐RP) (see also 13, 21, 22, 25, 46, 56). Third, twisting and pushing on the catheter resulted in a shift in the Pves pattern from PC‐RP to EO‐RP (Group 2 Experiment 3). The negative correlation between the Pves rebound amplitude and the bursting/ HPFO duration ratio in the EO‐PR (Figure 7(d)) suggests the longer the catheter tip remains obstructed, the more pressure is accumulated in the tubing that is still pumping saline. Additionally, the loss of the positive relation between the EUS burst duration and expelled urine volume for the EO‐PR voids (Figure 7(f)) suggests a diminution of the detrusor contraction efficiency that cannot be compensated by the pumping action of the EUS.

Taken together, the present results and those of others strongly suggest that rebound pressures that occur prior to EUS closure (EO‐RP) are artifact, likely from an obstruction of the catheter tip which can at times occur during contraction of the detrusor (Jin et al., 2014; Thor & Groat, 2010). A future imaging study (e.g., ultrasound and fluoroscopy) would further confirm this assertion. Thus, refinement of the CMG technique is recommended to ensure catheter stability so that the most accurate physiological CMG data can be collected. Note that the end of HFPOs is not a reliable way to determine urethral closure in continuous CMG performed using a single dome catheter. Closure of the urethra correlates best with the start of tonic electrical activity after bursting of the EUS. As already noted, the duration of HFPO and the bursting do not always match (Figures 3, 4(b2), and 5(b2)) (Chang et al., 2007; Salazar et al., 2019; Schneider et al., 2015; Zhang et al., 2008). While some studies that did not record EMG of the EUS report the expulsion of several drops during the rebound phase (Andersson et al., 2011; Jin et al., 2014; Lee et al., 2008; Maggi, Giuliani, et al., 1986; Maggi et al., 1986) without explanation, our results indicate that these leaks likely occur because the urethra is not yet closed as visualization of the HFPO is being masked by the pressure artifact. It is therefore also recommended that when EMG of the EUS is not possible, the duration of the void could be used to distinguish PC‐RP versus EO‐RP.

Two further findings during the emptying phase of the micturition cycle related more specifically to EUS function are also noteworthy. First, placement of a pressure transducer through the urethra produced an increase in EUS bursting duration. The increase of flow resistance caused by the urethral probe induced compensatory activity of the pumping action of the EUS to maintain void efficiency (Chen et al., 2012; LaPallo et al., 2014; Smith et al., 2008). Note that glutamatergic and serotonergic mechanisms are important in the reflex pathways underlying bladder‐sphincter coordination in rats (Chang et al., 2006; Chen, Fan, et al., 2012). Second, the duration of EUS bursting activity was positively correlated with void volume (Figure 6(e)). This finding is consistent with previous reports (Chen et al., 2013; LaPallo et al., 2016; Maggi, Giuliani, et al., 1986), and emphasizes the importance of the pump action of the sphincter for efficient bladder emptying. EUS bursting is an essential feature for efficient voiding in rodents (Cruz & Downie, 2005; Langdale & Grill, 2016), since the lack of this pumping action such as following spinal cord injury affects efficiency (D’Amico & Collins, 2012; Kakizaki et al., 1997; Kruse et al., 1993).

## 5 CONCLUSIONS

Taken together, the present results demonstrate that (1) urethane anesthesia has suppressive effects on the fill‐void cycle; (2) acute opening of the bladder wall for CMG assessments generates changes in detrusor contractile dynamics manifested as a decrease in the inter‐contractile interval, voided volume and the bursting time; (3) use of a single catheter to infuse fluid and measure the Pves modifies the shape of the pressure curve due to the resistance generated by the tube; and (4) use of a novel dual ureter configuration generates Pves recordings like those obtained with a single large catheter placed through the bladder dome, without any of the confounding variables. If the standard dome placement is used for CMG studies, it will be necessary to distinguish true rebound (PC‐RP) from artifact (EO‐RP) by determining the bursting‐HFPO ratio. If an EMG is not recorded, the duration of urine flow, recorded with a volume transducer, could be a good indicator of the opening and closure of the EUS. A flow duration that is longer than the HFPO would be indicative of an artificial rebound. If an artificial rebound phase pattern is suspected, it is recommended that the analysis exclude the third phase of CMG, the average voiding urine flow rate, and the closing pressure.

## DISCLOSURES

The authors declare that there is no conflict of interests.

## AUTHOR CONTRIBUTIONS

D.M.A. contributed to acquisition, analysis and interpretation of data, and drafted the manuscript. C.H.H. and J.L.Q. supervised the research and contributed to the acquisition, analysis, and interpretation of all data. R.F.H. and M.A.C. contributed to acquisition and interpretation of data. D.M.A., C.H.H., A.M., and J.L.Q. contributed to concept development and design. C.H.H. and J.L.Q. obtained funding. All authors critically reviewed and revised the manuscript.
